
JOURNAL INFORMATION
==============================
NLM Title Abbreviation: Behav Neurol
Journal ID: Behav Neurol
NLM Journal ID: 8914585
Journal ID: BN

Behavioural Neurology

ISSN: 0953-4180
EISSN: 1875-8584
Publisher: Wiley

ARTICLE INFORMATION
==============================
PMCID: PMC5215578
PMID: 22713425
DOI: 10.3233/BEN-2012-129008
Article ID: 915106
Article version: 1
Subjects: Other

Effectiveness of 6-Months Continuous Positive Airway Pressure Treactment in OSAS-Related Cognitive Deficit in Older Adults

Iglesias B. Gutiérrez 1 *
Escarceller C. Jacas 2
Robles I. Bardés 3
Masip R. Cambrodi 4
Santo-Tomás O. Romero 4
Navinés F. Pujadas 1
Rovira M. Boada 1
1 Unidad de Demencias Servicio de Neurología Hospital Universitario Vall d'Hebron Barcelona Spain
2 Neuropsicología Servicio de Psiquiatría Hospital Universitario Vall d'Hebron Barcelona Spain
3 Unidad de Geriatría Servicio de Medicina Interna Hospital Universitario Vall d'Hebron Barcelona Spain
4 Unidad Interdisciplinar de Sueño Servicio de Neurofisiología Clínica Hospital Universitario Vall d'Hebron Barcelona Spain
*B. Gutiérrez Iglesias: belengutierreziglesias@gmail.com
Print publication date: 2013
Volume: 26
Issue: 3
First page: 191
Last page: 194
Received 2012 May 21; Accepted 2012 May 21
Copyright: Copyright © 2013 Hindawi Publishing Corporation and the authors.
Copyright year: 2013
License: This is an open access article distributed under the Creative Commons Attribution License, which permits unrestricted use, distribution, and reproduction in any medium, provided the original work is properly cited.
License URL: https://creativecommons.org/licenses/by/3.0/

==============================
Behavioural Neurology 26 (2013) 191–194
DOI 10.3233/BEN-2012-129008
IOS Press

### Effectiveness of 6-months Continuous Positive Airway Pressure treactment in OSAS-related cognitive deficit in older adults

B. Gutiérrez Iglesias[a,*], C. Jacas Escarceller[b], I. Bardés Robles[c], R. Cambrodi Masip[d], O. Romero Santo-Tomás[d], F. Pujadas Navinés[a] and M. Boada Rovira[a]
[a]*Unidad de Demencias, Servicio de Neurología, Hospital Universitario Vall d'Hebron, Barcelona, Spain*
[b]*Neuropsicología, Servicio de Psiquiatría, Hospital Universitario Vall d'Hebron, Barcelona, Spain*
[c]*Unidad de Geriatría, Servicio de Medicina Interna, Hospital Universitario Vall d'Hebron, Barcelona, Spain*
[d]*Unidad Interdisciplinar de Sueño, Servicio de Neurofisiología Clínica, Hospital Universitario Vall d'Hebron, Barcelona, Spain*

### 1. Introduction

Obtructive Sleep Apnea Syndrome (OSAS) is a common disorder in ederly. The main symptoms reported by patients include: excessive daytime sleepiness (EDS), morning headaches, changes in mood and personality, cognitive problems and reduced quality of life.

Treatment of choice is CPAP (Continuous Positive Air Pressure), but findings on cognitive function are conflicting. Most authors have found improvement in certain cognitive functions. However some authors hold that evidences which support the potential benefit are weak due to the short treatment duration or the small sample.

### 2. Objectives

Few studies have followed patients older than 60 years or have administered a comprehensive neuropsychological battery. The aim of this study was to evaluate the effectiveness of CPAP in improving OSAS-related cognitive deficit in older adults.

*Corresponding author: B. Gutiérrez Iglesias, Unidad de Demencias, Servicio de Neurología, Hospital Universitario Vall d'Hebron, Barcelona, Spain. E-mail: belengutierreziglesias@gmail.com.

### 3. Material and methods

Thirteen patients with OSAS, diagnosed by polysomnography, were assessed prior to the initiation of CPAP treatment and were compared with 29 healthy controls. 6 month after treatment with CPAP, we compared OSAS patients performance with their baseline.

Inclusion criteria were: SAOS, age $>$ 60 years, normal MMSE, and not suffering from other chronic lung diseases, neurological or psychiatric illness. Because hypertension, dyslipidemia and diabetes are common in OSAS, these conditions were accepted along with the correct pharmacological treatment.

In the neuropsychological battery we included:

- Mini-Mental State examination (MMSE)
- Hospital Anxiety and Depression Scale (HAD)
- Short Form Medical Outcomes Survey (SF-36)
- Continuos Performance Test (CPT)
- Digits Span (Wais-III): Digits Span Forward (DSF) and Digits Span Backward (DSB)
- Spatial Span (WMS): Spatial Span Forward (SSF) and Backward (SSB)
- Trail Making Test, A and B (TMT)
- Symbol Digit Modality Test Oral (SDMTO)
- Stroop
- Tower of London (TOL)

ISSN 0953-4180/13/$27.50 © 2013 – IOS Press and the authors. All rights reserved

– Brown Peterson (BP)
– Paced Auditory Serial Addition Test (PASAT)
– Rey Auditory-Verbal Learning Test (RAVLT): Learning, Short Term (STM) and Long Term Memory (LTM)
– Rey-Osterreith Complex Figure Test (ROCFT)
– Grooved Pegboard Test

Nocturnal hypoxemia was assessed by analysis of the oxyhemoglobin saturation during sleep.

### 4. Results

OSAS patients obtained significantly lower scores ($p < 0.05$) at baseline than controls on: audioverbal span (DSF), audioverbal and visuospatial working memory (DSB, PASAT, SSB), alternating attention and cognitive flexibility (TMTB, Stroop 3), impulsivity and inhibition (Stroop 3, CPT), problem solving efficiency (TOL), processing speed (Stroop, PASAT, ROCFT time, TOL time) and dominant hand motor coordination (Grooved).

Six months after CPAP treatment, OSAS performance improved in all of them but differences are significant in a few tests: Divided attention and Working Memory (PASAT), Processing speed (PASAT and Stroop), problem solving (TOL), verbal learning and STM (RAVLT and BP).

### 5. Discussion

Performance of people with OSAS is significantly lower than control group except in sustained attention. Some authors obtained these results too. However most previous studies show that the OSAS group pays significantly worse in this role compared to healthy controls. We suggest that CPT may not be the test of choice, that sustained attention may be also altered in normal aging or simply we have a small sample. Our subjects improved in selective (CPT, TMTA and Stroop 3) and divided attention (PASAT).

Most authors suggest that memory deficit is due to difficulties of recovery while both storage and retrieve remain intact, showing that OSAS patients do not have the type of consolidation or storage problems seen in amnesic patients with temporal injuries and concluding that long-term memory deficits in OSAS are more related to memory disabilities associated with frontal lobe disturbances. Others suggest that memory impairment may be secondary to a deficit in storage associated to reduced oxygen levels with the involvement of the hippocampus, a structure that has been described as vulnerable in other diseases with hipoxemia. Our OSAS patients performed worse than controls on all facets of memory assessed. They showed a normal learning curve but with lower scores than the control group. It is not a typical pattern of hippocampal memory. Their performance improved significantly after treatment in learning, verbal STM and visual LTM.

In our study we evaluated executive functions extensively and all of them have been compromised in more or less degree. After CPAP we found that only improve TOL functions, such as problem-solving ability, motor speed, planning or impulsivity. However, we must beware of these results because we did not carry out a second evaluation to the control group and we cannot be sure that this improvement is not due to the practice effect.

Visuoconstruction is one of the few functions where OSAS and controls did not show significant differences in our study. A possible explanation could be that the test used to assess this function is not adequate.

OSAS patients in this research performed less than controls on motor coordination and, after CPAP, they remained impaired. To Aloia, this may suggest an irreversible damage to the basal ganglia, sensitive to repeated episodes of nocturnal hypoxemia.

In summary, OSAS-related cognitive abilities improved significantly after treatment in selective and divided attention, working memory, verbal learning and short-term memory and visual long-term memory. We did not find significant differences pre- and post-treatment, although we also observed an improvement over the baseline, in verbal long-term memory, cognitive flexibility, perseveration, processing speed or motor coordination (see Table 1).

Some authors have suggested that persistent cognitive impairments are related to the possibility of permanent brain damage. Periventricular white matter would be widely affected in OSAS, altering axons that connect structures. This can interfere with multiple aspects of cognitive and behavioral and, after several years without treatment, could generate a similar profile to a subcortical dementia.

Our study has some important limitations. First we have a relatively small number of subjects, so we have to be conservative with our findings. In addition, we have not carried out a second assessment to healthy controls, therefore, we cannot be ensure that improvements found after CPAP were due to treatment and not due to a practice effect.

Table 1
Pre and Post-treatment scores

| Test and cognitive function | PRE-T | POST-T | p |
|---|---|---|---|
| *Digits span (wais-III)* | | | |
| Digits span fordward (DSF) – *audio-verbal attentional span* | 6.9± 1.41 | 7.2± 2 | n.s. |
| Digits span backward (DSB) – *audio-verbal working memory* | 4.7+ 1.7 | 5+ 1.2 | n.s. |
| *Spatial span (WMS)* | | | |
| Spatial span fordward (SSF) – *visuospatial attentional span* | 7.2± 1.5 | 7.5± 1.9 | n.s. |
| Spatial span backward (SSB) – *visuospatial working memory* | 5.8± 0.93 | 5.9± 0.6 | n.s. |
| *Continuos performance test (CPT)* | | | |
| Selective attention | 17.8+ 22 | 11.8+ 13.5 | *0.05** |
| Inattention | 13± 8.5 | 18.6± 13.4 | n.s. |
| Sustained attention | −0.04± 0.06 | −0.04± 0.04 | n.s. |
| Impulsivity/planning | 3.5+ 8.6 | 1.8+ 2.1 | n.s. |
| Reacction time | 475.6± 130.5 | 443± 76 | n.s. |
| *Symbol digit modality test oral (SDMTO)* | | | |
| Correct – *Processing speed* | 35.2± 13.5 | 37.5± 12.9 | n.s. |
| Errors – *Selective attention* | 3.7+ 4.55 | 1.7+ 2.2 | n.s. |
| *Paced auditory serial addition test (PASAT)* | | | |
| 3” correct – *Working memory-IPS*[$] | 27.7± 14.2 | 33.4± 15.1 | n.s. |
| 3” errors – *Divided attention-IPS*[$] | 14.4± 13.6 | 5.7± 3.4 | n.s. |
| 2” correct – *Working memory-IPS*[$] | 20.3± 9 | 25.7± 12.4 | *0.036** |
| 2” errors – *Divived attention-IPS*[$] | 12.1+ 12.4 | 6.3+ 4.2 | n.s. |
| *Trail making test (TMT)* | | | |
| Trail making test A – Selective attention | 60.1± 16.9 | 56.8± 22.6 | n.s. |
| Trail making test A errors | 0.08+ 0.3 | 0n.s. | |
| Trail making test B – *Alternanting attention* | 230± 164 | 181± 133 | n.s. |
| Trail making test B errors – *Cognitive flexibility* | 2.2+ 2.4 | 1.3+ 1.8 | n.s. |
| *STROOP* | | | |
| Stroop 1 – *Information processing speed* | 76.8± 18.6 | 79.3± 19.8 | n.s. |
| Stroop 2 – *Information processing speed* | 49.9± 11.9 | 55.2± 10.4 | *0.041** |
| Stroop 3 – *Selective attention* | 29± 7.8 | 32.3± 9.2 | *0.035** |
| Stroop 3 errors – *Inhibition* | 1.2+ 1.3 | 0.9+ 0.9 | n.s. |
| *Tower of london (TOL)* | | | |
| Total correct – *Problem solving capacity* | 2.9± 2.2 | 4.9± 2 | *0.007** |
| Total moves – *Problem solving efficiency* | 49.1± 22.8 | 36.6± 21.3 | *0.016** |
| Total iniciation time – *Planning/impilsivity* | 94.5± 66 | 74.7± 32.5 | n.s. |
| Total execution time – *Executive speed* | 374± 196 | 298± 183 | *0.006** |
| Total time – *Problem solving time* | 470± 212 | 372± 190 | *0.006** |
| Total time violation – *Slow cognitive processing* | 2.8± 2.3 | 1.8± 2 | *0.009** |
| Total rukes violation – *Capacity to mantain a complex action* | 1.8+ 2 | 0.8+ 1 | n.s. |
| *Brown peterson task* | | | |
| Total – *Short time memory* | 36.54± 8 | 14.3± 29 | *0.035** |
| Perseverations | 5± 3.1 | 6.6+ 4 | n.s. |
| *Rey auditory-verbal learning test (TAVLR)* | | | |
| Learning | 35.3± 11.4 | 40.2± 8.6 | *0.024** |
| Short term (STM) | 7± 3.3 | 8.2± 2.6 | *0.032** |
| Long term memory (LTM) | 6.8± 4.1 | 7.6± 2.9 | n.s. |
| Recognition | 10.5± 3.1 | 11.6± 2.4 | n.s. |
| *Rey-osterreith complex figure test (ROCFT)* | | | |
| Copy time – *Speed* | 484± 365 | 356± 134 | n.s. |
| Copy – *Visuoconstruction* | 32± 2.4 | 32.7± 2 | n.s. |
| *Short term (STM)* | 16.7± 5 | 19.5± 6 | n.s. |
| *Long term memory (LTM)* | 15.1± 5.9 | 18.4± 6.3 | *0.064*[T] |
| *Recognition* | 19.2± 3.2 | 19.5± 1.8 | n.s. |
| *Grooved pegboard test* | | | |
| Dominant hand – *dominant hand motor coordination* | 83.9± 15.7 | 79.4± 16.1 | n.s. |
| No dominant hand – *No dominant hand motor coordination* | 109± 60.3 | 100± 46 | n.s. |

[*]Statístical significant difference pre- and post-treatment [T]Trend toward significant difference [$]Information Processing Speed.

### 6. Conclusions

Our findings provide evidence for clinically significant benefits to some OSAS-related cognitive abilities in older adults. However we cannot associate this improvement with any particular cognitive domain.

### References

[1] M. Aloia, J.T. Arnedt, J.D. Davis, R.L. Riggs and D. Byrd, Neuropsychological sequelae of obstructive apnea-hypopnea syndrome, *J Int Neuropsychol Soc* **10** (2004), 772–785.

[2] M.S. Aloia, N. Ilniczky, P. Di Dio, M.L. Perlis, D.W. Greenblatt and D.E. Giles, Neuropsychological changes and treatment compliance in older adults with sleep apnea, *J Psychosom Res* **54** (2003), 71–76.

[3] M. Aloia, Neuropsychological consequences of Obstructive Sleep Apnea. Considerations for treatment. Bussiness Brielfing: US Respiratory Care, 2005.

[4] R.L. Cross, R. Kumar, P.M. Macey, L.V. Doering, J.R. Alger, F.L. Yan-Go and R.M. Harper, Neural Alterations and Depressive Symptoms in Obstructive Sleep Apnea Patients, *Sleep* **31**(8) (2008).

[5] A. Décary, I. Rouleau and J. Montplaisir, Cognitive deficits associated with sleep apnea syndrome: a proposed neuropsychological test battery, *Sleep* **23**(3) (1 May 2000), 369–381. Review.

[6] B. El-Ad and P. Lavie, Effect of sleep apnea on cognition and mood, *Int Rev Psychiatry* **17**(4) (Aug 2005), 277–282. Review.

[7] K.A. Felmet and M. Petersen, Obstructive sleep apnea and cognitive dysfunction, *JAAPA* **19**(11) (Nov 2006), 16–20. Review.

[8] L. Ferini-Strambi, C. Baietto, M.R. Di Gioia et al., Cognitive dysfunction in patients with obstructive sleep apnea (OSA): partial reversibility after continuous positive airway pressure (CPAP), *Brain Res Bull* **61** (30 2003), 87–92.

[9] W. Lim, W.A. Bardwell, J.S. Loredo, E. Kim, S. Ancoli-Israel, E.E. Morgan, R. Heaton and J.E. Dimsdale, Effects of two-weeks continuous positive airway pressure treatment vs. oxygen supplementation on neuropsychological functioning in patients with obstructive sleep apnea, *Clinical Sleep Medicine* **3**(4) (2007), 380–386.

[10] C. Monasterio, S. Vidal, J. Durán, M. Ferrer, C. Carmona, F. Barbé, M. Mayos, N. González-Mandago, M. Juncadella, A. Navarro, R. Barreira, F. Capote, L.R. Mayoralas, G. Peces-Barba, J. Alonso and J.N. Montserrat, Effectiveness of continuous positive airway pressure in mild sleep apnea–hypopnea syndrome, *Am J Respir Crit Care Med* **164** (2001), 939–943.
